# Severity and Duration of Acute Kidney Injury and Chronic Kidney Disease after Cardiac Surgery

**DOI:** 10.3390/jcm10081556

**Published:** 2021-04-07

**Authors:** Suk Hyung Choe, Hyeyeon Cho, Jinyoung Bae, Sang-Hwan Ji, Hyun-Kyu Yoon, Ho-Jin Lee, Ji-Hyun Lee, Jin-Tae Kim, Won Ho Kim

**Affiliations:** Department of Anesthesiology and Pain Medicine, Seoul National University Hospital, Seoul National University College of Medicine, #101 Daehak-ro, Jongno-GU, Seoul 03080, Korea; paulchoe17@snu.ac.kr (S.H.C.); bdbd7799@gmail.com (H.C.); baejy88@gmail.com (J.B.); taepoongshin@gmail.com (S.-H.J.); hyunkyu18@gmail.com (H.-K.Y.); zenerdiode03@gmail.com (H.-J.L.); muslab@hanmail.net (J.-H.L.); jintae73@gmail.com (J.-T.K.)

**Keywords:** acute kidney injury, chronic kidney disease, risk factor, duration, creatinine, cardiac surgery

## Abstract

We aimed to evaluate whether the duration and stage of acute kidney injury (AKI) are associated with the occurrence of chronic kidney disease (CKD) in patients undergoing cardiac or thoracic aortic surgery. A total of 2009 cases were reviewed. The patients with postoperative AKI stage 1 and higher stage were divided into transient (serum creatinine elevation ≤48 h) or persistent (>48 h) AKI, respectively. Estimated glomerular filtration rate (eGFR) values during three years after surgery were collected. Occurrence of new-onset CKD stage 3 or higher or all-cause mortality was determined as the primary outcome. Multivariable Cox regression and Kaplan–Meier survival analysis were performed. The Median follow-up of renal function after surgery was 32 months. The cumulative incidences of our primary outcome at one, two, and three years after surgery were 19.8, 23.7, and 26.1%. There was a graded significant association of AKI with new-onset CKD during three years after surgery, except for transient stage 1 AKI (persistent stage 1: HR 3.11, 95% CI 2.62–4.91; transient higher stage: HR 4.07, 95% CI 2.98–6.11; persistent higher stage: HR 13.36, 95% CI 8.22–18.72). There was a significant difference in survival between transient and persistent AKI at the same stage. During three years after cardiac surgery, there was a significant and graded association between AKI stages and the development of new-onset CKD, except for transient stage 1 AKI. This association was stronger when AKI lasted more than 48 h at the same stage. Both duration and severity of AKI provide prognostic value to predict the development of CKD.

## 1. Introduction

Chronic kidney disease may develop after open cardiac surgery [1] or coronary artery bypass graft [2]. End-stage renal disease contributes to increased hospitalization, poor quality of life, and the development of comorbidities, including ischemic heart disease, cerebrovascular accident, and frailty [3,4,5]. However, the overall incidence and risk factors for the development of chronic kidney disease (CKD) after open cardiac or thoracic aortic surgery have not been evaluated fully.

It has been reported that acute kidney injury (AKI) is an important contributor to postoperative morbidity and mortality after cardiovascular surgery [6,7,8]. The incidence of AKI after cardiovascular surgery is as high as 55% [7,9,10,11,12,13], and temporary deterioration of renal function after aortic surgery is related to higher long-term mortality [14]. Although the associations between AKI and CKD were reported in various medical and surgical populations [15,16], this relationship has been rarely reported after cardiac surgery [2,17]. Particularly, the association between the severity of postoperative AKI and the risk of the occurrence of new-onset CKD after cardiovascular surgery has been rarely reported. A previous study reported the duration of postoperative AKI is related to long-term survival after cardiac surgery [18]. However, the relationship between the duration of AKI and CKD after cardiac surgery is still unknown, and there has been no previous study evaluating whether persistent elevation in serum creatinine is associated with the risk of developing chronic kidney disease after cardiac surgery. It is important to find risk factors of the long-term renal dysfunction or the occurrence of CKD after cardiac surgery because perioperative management of the patients at high risk for long-term renal dysfunction may prevent further renal functional decline and improve patient prognosis.

Therefore, in this retrospective observational cohort study, we sought to evaluate the long-term renal function in patients undergoing cardiac or thoracic aortic surgery. We hypothesized that the patients with postoperative AKI with long duration, i.e., persistent AKI would be at higher risk to develop CKD, compared to transient AKI. Additionally, we tested the hypothesis that the persistent AKI of the same stage is more strongly associated with CKD than the transient AKI of the same stage. Additionally, we evaluated whether the transient AKI of stage 1 may have the same risk of developing CKD as the patients with no AKI.

## 2. Materials and Methods

### 2.1. Study Design

The institutional review board (IRB) of Seoul National University Hospital approved this study (1909-115-1066) on 30 September 2019. Written informed consent was waived by the IRB given the retrospective design of our study. Our study protocol was registered at www.clinicaltrials.gov prior to data collection and analysis (NCT04172103, first posted on 21 November 2019). We reviewed the electronic medical records of 2510 consecutive adult patients who underwent cardiac surgery or surgery on the thoracic aorta under cardiopulmonary bypass (CPB) at our hospital between 2008 and 2017. The patients with missing baseline serum creatinine (sCr) value (*n* = 70), missing postoperative sCr values of more than three among seven days (*n* = 128), baseline CKD with estimated glomerular filtration rate (eGFR) < 60 mL/min/1.73 m^2^ (*n* = 98), and off-pump coronary artery bypass (*n* = 355) were excluded. As a result, a total of 2009 patients were included in our analysis (Supplemental Figure S1).

### 2.2. Surgical Procedures and Anesthetic Management

Valve replacement surgery and coronary artery bypass surgery were performed under CPB with moderate hypothermia. Thoracic aortic surgery was performed under CPB with moderate or deep hypothermia. Anesthesia was maintained with a target-controlled infusion of propofol and remifentanil. Radial and femoral arterial pressure were continuously monitored. A pulmonary artery catheter was placed to monitor continuous cardiac output. Vasopressor including norepinephrine or phenylephrine and inotropes including dobutamine or epinephrine were administered, guided by monitored cardiac output, mixed venous oxygen saturation, and systemic vascular resistance.

### 2.3. Data Collection and Study Outcomes

Demographic or perioperative variables reported to be associated with postoperative renal dysfunction in previous studies were collected by reviewing electronic medical records (Table 1) [9,10,11,12,13,19,20,21,22,23,24,25,26]. We defined postoperative AKI and its stages using the Kidney Disease Improving Global Outcomes (KDIGO) serum creatinine criteria [27]. AKI was classified into three stages as follows: Stage 1: ≥0.3 absolute sCr increase within postoperative 48 h or ≥50% relative sCr increase within the seven postoperative days; stage 2: ≥100% relative sCr increase within the seven postoperative days; stage 3: ≥200% relative sCr increase within the seven postoperative days. Patients were classified based on the highest stage evaluated during the postoperative seven days. 

The baseline sCr was defined as the most recent measured sCr before surgery [28]. Transient AKI was defined when sCr increase to meet the sCr criteria of the highest stage was maintained for ≤48 h, and persistent AKI was defined when sCr increase to satisfy the sCr criteria of AKI for >48 h [29]. Each stage of AKI was further classified into transient and persistent AKI. Given their relatively low incidence, stage 2 and 3 AKI were considered to be a single group of stage 2 or 3 AKI. As a result, there are five groups in our study—no AKI, transient stage 1 AKI, persistent stage 1 AKI, transient stage 2 or 3 AKI, and persistent stage 2 or 3 AKI.

The primary outcome of the study was the development of new-onset CKD stage 3 or higher or all-cause mortality during the three years after surgery. The new-onset CKD was defined by at least two consecutive eGFR values < 60 mL/min/1.73 m^2^, separated by an interval of at least three months [30,31]. The eGFR values were considered to be measured at the specific time points when the values were measured within one month before or after our pre-specified time points. The eGFR values were estimated using the modification of diet in renal disease (MDRD) study equation [32]. When the renal function recovered from CKD stage 3 thereafter, the case was excluded from our primary outcome. However, the recovered case remained in the incidence of eGFR values < 60 mL/min/1.73 m^2^ at three months, one, two, and three years after surgery before their renal function recovered.

The secondary outcomes included the incidence of new-onset stage 3 or higher CKD or all-cause mortality during one and two years after surgery, and the incidence of dependence on the regular hemodialysis. Postoperative other secondary clinical outcome variables included length of postoperative hospital or intensive care unit (ICU) stay, in-hospital all-cause mortality, intra- or postoperative intra-aortic balloon pump (IABP) insertion, reopen for surgical bleeding, and postoperative respiratory complications. Respiratory complications included postoperative atelectasis, pleural effusion, pneumothorax, pneumonia, and pulmonary edema.

### 2.4. Statistical Analysis

STATA/MP version 15.1 (StataCorp, College Station, TX, USA) was used to analyze the data. For all analyses, *p* < 0.05 was considered statistically significant. The normality of the data distribution was determined by visual inspection of quantile–quantile plot. Continuous variables are described as the median (25 and 75 percentiles) and were compared with the Mann–Whitney U test. Categorical variables were compared with Fisher’s exact test or χ2 test according to the expected counts. To evaluate the potential selection bias caused by our inclusion criteria, we compared the baseline characteristics between the included and excluded patients. Missing data were present in less than 8% of the baseline and surgery-related variables of Table 1. There was no missing data in our primary outcome because those with missing outcomes were excluded according to our inclusion criteria. The frequency of cases without missing data was reported for all variables in Table 1. For potential outliers, one of our investigators verified in our source document and excluded definite outliers from our analysis.

The following statements are the summary of our main statistical analyses. Firstly, to evaluate the association between severity and duration of AKI and our primary outcome, multivariable Cox regression analysis was performed. Proportional hazard assumptions for categorical variables were evaluated by visual inspection of log minus–log survival plots and those for continuous variables were tested by restricted cubic splines for continuous variables [33,34]. All baseline and surgery or anesthesia-related parameters were considered as covariates. We evaluated the multicollinearity among covariates before conducting multivariable analysis by using the variance inflation factor. For the complete case analysis, we excluded the cases with missing values of the covariates from the Cox regression analysis. We excluded a variable with a variance inflation factor greater than five from our analysis. The stepwise variable selection process with the backward Wald method using a significance cutoff of 0.20 was performed. We evaluated the calibration of Cox regression model by Gronnesby and Borgan test [35]. We measured the discrimination of the Cox regression model by Harrell’s C and Somer’s D [36].

Secondly, to evaluate the interaction between severity and duration of AKI with the time-dependent development of all-cause mortality or new-onset CKD after surgery, Kaplan–Meier survival curve analysis was performed between the five AKI-related groups including no AKI, transient stage 1 AKI, persistent stage 1 AKI, transient stage 2 or 3 AKI, and persistent stage 2 or 3 AKI. Kaplan–Meier analysis was performed because our primary endpoint was a time-to-event outcome, and follow ups of renal function were right censored. A log-rank test was used to compare overall survival between groups.

Thirdly, the secondary clinical outcomes of our study were compared between different AKI stages, including transient and persistent AKI groups. One-way ANOVA was used to compare the incidence of outcomes between different AKI groups.

Fourthly, the absolute eGFR values and cumulative incidences of new-onset CKD were compared at three months, one, two, and three years after surgery by Mann–Whitney U test and chi-square test, respectively. Bonferroni correction was used to adjust multiple comparisons, and *p* < 0.013 was considered to be significant. The interactions between the in eGFR and time were compared between different AKI groups by two-way analysis of variance (ANOVA).

Fifthly, we drew cubic spline function curves to evaluate the possible linear or curvilinear relationship between the duration of AKI stage 1 or stage 2 or 3 and the risk of new-onset CKD or all-cause mortality during three years after surgery.

Sixthly, for a sensitivity analysis to evaluate the association of other cutoffs of persistent AKI with our primary outcome, we performed multivariable Cox regression analysis using AKI with a duration of between 7 and 90 days. The cutoff of seven days was determined according to the concept of acute kidney disease [29]. All covariates of our main Cox regression analyses were included in this sensitivity analysis again. To compare the survival between the patients with and without AKI with a duration of more than seven days, Kaplan–Meier survival curve analysis was performed again.

Finally, as a post hoc analysis, given the persistent stage 2 or 3 AKI is the strongest risk factor of our primary outcome, we performed multivariable logistic regression analysis for persistent stage 2 or 3 AKI. All covariates of our Cox regression analysis except AKI stages were used. The stepwise variable selection process with the backward Wald method using a significance cutoff of 0.20 was performed.

With our available sample size of 1356 patients used for Cox regression analysis, we had 99% power to detect a priori hazard ratio, which we deemed clinically important with a significance criterion of 0.05. For this calculation, we hypothesized the hazard ratio of persistent AKI stage 1 was 2.00 for our primary outcome by proportional hazard regression analysis. The probability of events and censoring was assumed to be 0.45. The smallest effect size, which we had 90% power to detect with 1356 patients, was a hazard ratio of 1.30.

## 3. Results

Patient characteristics and perioperative parameters are summarized in Supplemental Table S1. These characteristics are compared between different AKI groups in Table 1. Supplemental Table S2 compares baseline characteristics and perioperative parameters between the patients with and without new-onset CKD or all-cause mortality during three years after surgery. The characteristics of the patients who were included and excluded from our study were compared in Supplemental Table S3. There was no significant difference between the included and excluded patients.

Median (interquartile range) follow up of renal function after surgery was 32 (12–37) months. Among 2009 patients who had postoperative sCr values, 652 patients (32.5%) were diagnosed with postoperative AKI using KDIGO criteria that was further categorized as transient stage 1 (*n* = 388, 19.3%), persistent stage 1 (*n* = 143, 7.1%), transient stage 2 or 3 (*n* = 58, 2.9%), and persistent stage 2 or 3 (*n* = 63, 3.1%) according to 48 h of AKI duration. The number of patients with AKI lasting more than seven days was 4.4% (*n* = 88, stage 1) and 1.6% (*n* = 32, stage 2 or 3).

Among the 2009 patient included in our analysis, 83.9% (*n* = 1685), 67.9% (*n* = 1364), and 60.1% (*n* = 1208) patients had follow-up sCr values at one, two, and three years after surgery. The cumulative incidence of new-onset CKD during one, two, and three years after surgery were 19.8% (*n* = 334/1685), 23.7% (*n* = 323/1364), and 26.1% (*n* = 315/1208). The cumulative incidence of new-onset CKD stage 3 or higher or all-cause mortality were 24.5% (*n* = 428/1789), 30.2% (*n* = 450/1491), and 34.1% (*n* = 463/1356). The cumulative incidence of the dependence on the regular hemodialysis at three years after surgery was 4.6% (*n* = 56/1208).

Table 2 shows the results of multivariable Cox regression analysis for the new-onset CKD or all-cause mortality during three years after surgery. Transient stage 1 AKI was not significantly associated with CKD (hazard ratio (HR) 1.95, 95% confidence interval (CI) 0.83–3.02, *p* = 0.246). However, persistent stage 1 AKI, transient and persistent stage 2 or 3 AKI showed significant and graded associations with the risk of CKD (persistent stage 1: HR 3.11, 95% CI 2.62–4.91; transient higher stage: HR 4.07, 95% CI 2.98–6.11; persistent higher stage: HR 13.36, 95% CI 8.22–18.72, all *p* < 0.001). The calibration of our Cox regression model was good (Gronnesby and Borgan test: χ2 = 0.748, *p* = 0.421). Harrell’s C was 0.32 and Somer’s D was 0.29. The results of Cox regression analyses for CKD during one and two years after surgery are shown in Supplemental Tables S4 and S5. The graded associations were also found between the stage and duration of AKI and our primary outcome.

**Figure 1:**
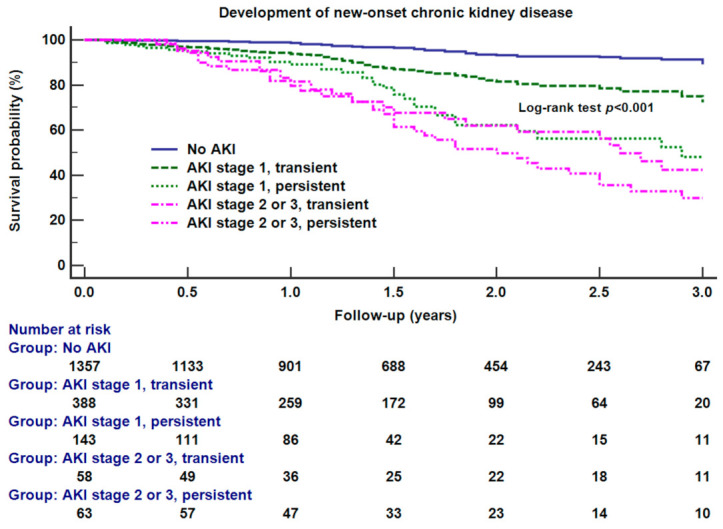
Kaplan–Meier survival curve analysis between different groups of acute kidney injury (AKI), i.e., no AKI, transient stage 1, persistent stage 1, transient stage 2 or 3, persistent stage 2 or 3. The results of the overall log-rank test were shown in the figure. There were significant differences in survival between transient and persistent stage 1 AKI (log-rank test *p* = 0.002) and between transient and persistent stage 2 or 3 AKI (log-rank test *p* = 0.010). The transient stage means that the increase in serum creatinine above the cutoff of serum creatinine criteria of acute kidney injury lasts less than 48 h, and the persistent stage means that creatinine increase lasts longer than 48 h.

Figure 1 shows the results of Kaplan–Meier survival curve analysis between different transient and persistent AKI groups. Overall, there were significant differences between groups (*p* < 0.001). There were significant differences between no AKI and transient stage 1 AKI (log-rank test *p* = 0.009), transient and persistent stage 1 AKI (log-rank test *p* = 0.010), or transient and persistent stage 2 or 3 AKI (log-rank test *p* = 0.021).

Table 3 shows the comparisons of our secondary outcomes between different AKI groups of stage and duration. There were significant differences between the transient and persistent AKI groups of the same stage regarding the length of postoperative hospital stay, ICU stay, complication rate, and in-hospital mortality rate.

Supplemental Figure S2 shows the comparison of the time-dependent changes in eGFR between the patients without AKI, those with transient or persistent stage 1 AKI, and those with transient or persistent stage 2 or 3 AKI. There were no significant differences between transient and persistent AKI groups in the same stages at the same time points (three years: stage 1 transient vs. persistent, *p* = 0.040; stage 2 or 3 transient vs. persistent, *p* = 0.032). Two-way ANOVA showed that there was no significant interaction between time and duration of AKI in the same stage (stage 1: *p* = 0.125; stage 2 or 3: *p* = 0.452). The cumulative incidences of eGFR 30–59, 15–29, and <15 mL/min/1.73 m^2^ were shown in Supplemental Figure S3.

**Figure 2:**
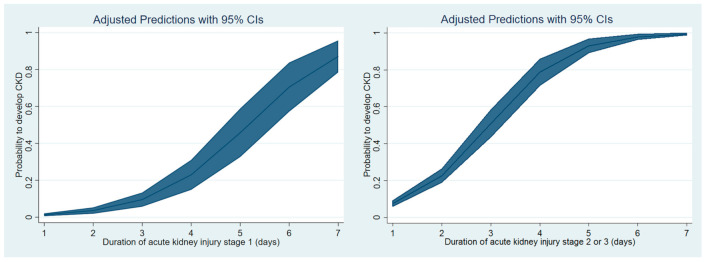
Cubic spline function curves of the relationship between the duration of acute kidney injury and the probability of developing chronic kidney disease as determined by estimated glomerular filtration rate of less than 60 mL/h/1.73 m^2^. CKD = chronic kidney disease.

Figure 2 shows that cubic spline function curves showing the relationship between the duration of AKI and the probability of developing CKD. There was a curvilinear positive relationship for both stage 1 and stage 2 or 3 AKI. An inflection point appears to exist for stage 2 or 3 AKI and the risk of CKD increases steeply when the duration of AKI was three days or more.

Supplemental Table S6 shows the results of multivariable Cox regression analysis for our primary outcome using the cutoff of AKI duration of more than seven days. The association was stronger between persistent AKI using a cutoff of seven days and our primary outcomes compared to persistent AKI using a cutoff of 48 h (AKI stage 1, more than seven days: HR 3.85, 95% CI 2.89–5.63, *p* < 0.001; AKI stage 2 or 3: more than seven days: HR 15.75, 95% CI 9.42–24.19, *p* < 0.001).

Supplemental Table S7 shows the results of the multivariable logistic regression analysis for persistent stage 2 or 3 AKI. Age, hypertension, preoperative hematocrit, combined procedure, intraoperative red blood cell transfusion, and epinephrine infusion were identified as significant risk factors.

**Figure 3:**
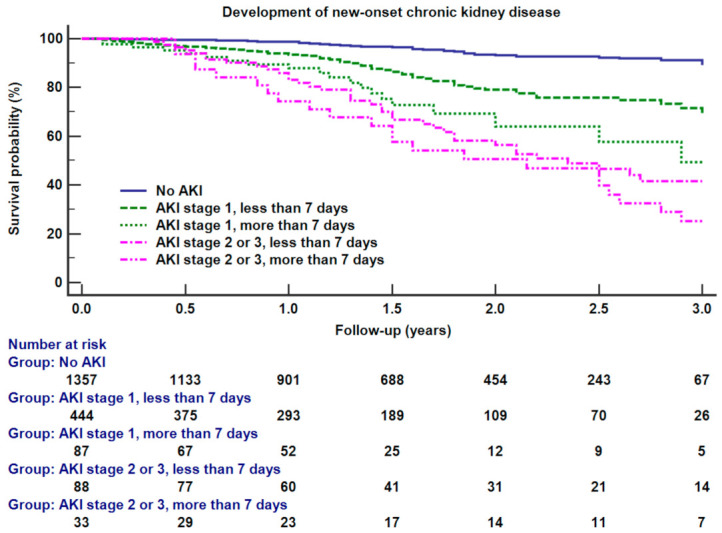
Kaplan–Meier survival curve analysis between different groups of acute kidney injury (AKI), i.e., no AKI, AKI stage 1 lasting less than seven days, AKI stage 1 lasting more than seven days, AKI stage 2 or 3 lasting less than seven days, AKI stage 2 or 3 lasting more than seven days. There were significant differences in survival between different AKI groups (log-rank test *p* < 0.001).

Figure 3 shows Kaplan–Meier survival curve analysis between different AKI groups according to its duration of seven days. There were significant differences in survival between groups (log-rank test *p* < 0.001). There were significant differences in survival between no AKI and stage 1 AKI with less than seven days (log-rank test *p* = 0.009), stage 1 AKI with less and longer than seven days (log-rank test *p* = 0.015), or stage 2 or 3 AKI with less and longer than seven days (log-rank test *p* = 0.046).

## 4. Discussion

We investigated the association between the duration of AKI and long-term renal function after cardiac or aortic surgery. The incidence of stage 3 or high CKD during three years after surgery was 26.1%. There was a significant difference in the survival between transient and persistent AKI, even in the same stage of AKI. Persistent stage 1 AKI was also significantly associated with new-onset CKD or all-cause mortality, while transient stage 1 AKI was not significantly associated with CKD. Persistent higher stage AKI was more strongly associated with CKD, compared to transient higher stage AKI. Our study results consolidate the notion that AKI is a strong risk factor for the development of CKD and that both the duration and severity of AKI are important for that association. We also demonstrated AKI lasting more than seven days is more strongly associated with our primary outcome, suggesting the importance of AKI lasting more than a week.

To our knowledge, only a few previous studies investigated the risk factors of new-onset CKD after cardiac or thoracic aortic surgery [2,17,37]. A previous retrospective cohort study also reported that AKI is a significant predictor of CKD after cardiac surgery [17]. This study developed a practical score to predict CKD after cardiac surgery based on the multivariable regression model [17]. They initially excluded the patients with stage 3 or high CKD. The significant risk factor of CKD included baseline renal function, old age, transplantation or aortic surgery, long aortic clamp time, and postoperative AKI [17]. High stages of AKI were more strongly associated with CKD than stage 1 AKI, which is consistent with our study. However, type of surgery was not a significant predictor in our study, which may be due to the small number of aortic surgery included in our data. Old age and baseline renal function were also significant in our study [2,17]. In addition, body mass index and history of diabetes mellitus were also significant in our study; both are known risk factors of CKD in the general population [25,26]. Another retrospective study also reported a prediction model for the end-stage renal disease after coronary artery bypass surgery [2]. In their multicenter retrospective cohort with a median follow-up of six years, they did not exclude the patients with baseline CKD and reported that both baseline postoperative AKI and preoperative CKD were significant predictors of CKD.

Legouis et al. reported the association between reversible AKI and CKD after cardiac surgery [37]. They compared the incidence of new-onset CKD between the patients with a fully recovering AKI and those without AKI in a bootstrapped matched cohort. They defined the “fully recovering AKI” when eGFR recovered to ≥90% of baseline and eGFR was ≥60 mL/min/1.73 m^2^ during the 7 to 90 days after surgery. Even fully recovering AKI was significantly associated with the increased risk of CKD, suggesting the importance of the renal functional follow-up in patients who developed AKI after cardiac surgery.

The relationship between AKI and CKD in the general medical population has been consistently reported [15]. AKI and CKD are considered to be interconnected and likely represent a continuum. A patient with a sustained episode of AKI has an increased risk of developing a new-onset CKD or worsening of pre-existing CKD. The duration of AKI was categorized into three groups of 1–2 days, 3–6 days, and ≥7 days and showed graded association with mortality during five years after cardiac surgery [18]. A previous acute disease quality initiative (ADQI) workgroup reported that persistent AKI frequently progresses to acute kidney disease [29]. Acute kidney disease is a proposed concept to characterize the course of disease after AKI, which exists between AKI and CKD [29,30]. Acute kidney disease was defined when AKI stage 1 or higher is present ≥7 days after an AKI initiating event such as surgery. ADQI workgroup suggested the optimal duration of persistent AKI reversal to be more than 48 h, which was used in our study. The workgroup explained the reason why the cutoff of 48 h rather than 72 h was used to define rapid reversal of AKI was to sensitively and quickly identify high-risk patients for whom additional evaluation is required [29]. Our study also demonstrated that the risk of CKD is even higher when AKI lasts longer than seven days even in stage 1 AKI. Further research is critical to delineate the relationship between AKI, acute kidney disease (AKD), and CKD after cardiac surgery and find modifiable risk factors to decrease the risk of CKD.

In our study, transient stage 1 AKI was not significantly associated with the development of CKD in our Cox regression analysis. However, Kaplan–Meier analysis showed a significant difference in renal survival between transient stage 1 AKI and no AKI. We defined transient stage 1 AKI as the complete reversal of AKI within 48 h of AKI onset. These results suggest that even the transient stage 1 AKI has significant prognostic importance in the development of CKD or long-term renal functional decline.

Our results could help the physician to differentiate the risk of AKI to develop long-term renal functional decline and plan a proper renal follow-up schedule to focus on the higher-risk patients to develop long-term renal dysfunction. Renal function of the patients who developed transient AKI should be followed up on the third day after the occurrence of AKI to monitor whether persistent AKI develops. Additionally, renal function of those with persistent AKI should be followed up seven days after the onset of AKI to evaluate whether acute kidney disease develops.

According to our sensitivity analysis, AKI with a duration of seven days or more was associated with the development of chronic kidney disease more strongly than AKI with a shorter duration. Recently, ADQI 16 workgroup proposed the concept of acute kidney disease, which is characterized by the continuance of AKI by sCr or urine output criteria for more than seven days [29]. Persistent AKI develops in a subset of patients with AKI, and the presence of persistent AKI or acute kidney disease should be regarded as an alarm to initiate a further evaluation of renal function and treatment options. Our sensitivity analysis supports the concept of acute kidney disease in cardiac surgical patients and highlighted the importance of long-term follow-up of renal function in patients with AKI.

**Figure 4:**
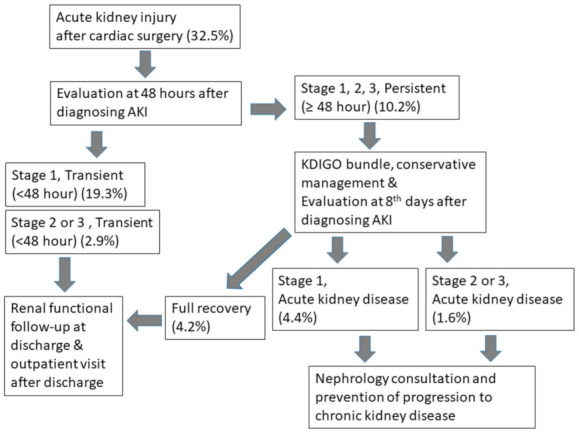
Cubic spline function curves of the relationship between the duration of acute kidney injury and the probability of developing chronic kidney disease, as determined by estimated glomerular filtration rate of less than 60 mL/h/1.73 m^2^. CKD = chronic kidney disease.

We developed a flow diagram for stepwise renal functional follow up in patients who developed AKI after cardiac surgery (Figure 4). We should follow up renal function even in transient AKI because transient AKI is significantly associated with renal survival. For persistent AKI, efforts to prevent progression to chronic kidney disease. Although it has not been established, the following were suggested for initial management of persistent AKI by the ADQI workgroup: reassessment of underlying etiology of AKI, optimization of hemodynamic and volume status, and conservative management such as hemodialysis to correct fluid overload, acidosis, and hyperkalemia. We should check the renal function on the eighth day after the onset of AKI to evaluate whether AKDhas developed. The timely assessment of renal function is important for consulting the patients with persistent AKI or acute kidney disease to nephrologists for adequate management and prevention of chronic kidney disease.

Recently, ADQI published the quality improvement goals to provide high-quality care for patients who experienced AKI [38]. In the schema for AKI and AKD follow up, they suggested the bundle care of kidney function check, advocacy, medications, pressure, and sick day (KAMPS) and weight assessment, access, teaching, clearance, hypotension, and medications (WATCH-ME) protocols according to the stage and duration of AKI and AKD. The group suggested detailed outlines of the patient care in each phase of AKI and AKD severity with these kidney health care bundles of KAMPS and WATCH-ME. These quality improvement goals should be checked during the follow up of the patients with AKI or AKD.

There are several important limitations of our study. Firstly, our study has the inherent limitations associated with a single-center retrospective analysis with missing renal functional follow up and possible information bias by possible inaccuracy in medical records. Our patients may not represent general cardiac surgical patients. Additionally, during the long-term follow up, patients may administer drugs that may impair their renal function. Furthermore, a detailed evaluation of recovery of renal function could not be performed, and our estimation of the incidence of CKD could be overestimated. However, we found no significant difference between the baseline characteristics of included and excluded patients. Nonetheless, unknown or unmeasured covariates may influence our results. Secondly, we could not use the urine output criteria to define AKI because hourly urine output was not measured during the postoperative seven days. Additionally, previous studies reported that the cutoffs of urine output criteria may be different in the surgical setting especially during cardiac surgery with CPB [39,40]. Urine output criteria are also regarded as less reliable in predicting AKI after surgery because oliguria could develop simply due to decreased preload in addition to intrinsic renal dysfunction [41]. Thirdly, the incidence of respiratory complications was only 17.5% in stage 2 or 3 persistent AKI, which was lower than that of stage 1 AKI. This is unusual and seems to be caused by data collection bias by retrospective data collection and omission of recording respiratory complication in progression note or chest X-ray reading.

## 5. Conclusions

We found a significant graded association between the stages or duration of AKI and the development of new-onset CKD or all-cause mortality after cardiac or thoracic aortic surgery. Specifically, there was a significant difference in renal survival between transient and persistent AKI. We also found a similar significant difference in survival between AKI with less than or longer than seven days. These differences were also found across different stages of AKI. The renal functional decline during three years after surgery was influenced by both duration and stages of AKI. Our results suggest that both severity and duration of AKI have important prognostic value to predict long-term renal functional impairment. These two aspects of AKI should be considered to guide the patients for postoperative renal functional follow up. Patient management strategies for persistent AKI and acute kidney disease should be investigated to mitigate the risk of CKD after cardiac surgery. Renal functional follow up and early consultation with a nephrologist are strongly recommended for patients who developed persistent AKI or CKD after cardiac surgery. Our data of long-term renal functional follow-up after cardiac surgery would be helpful to develop a guidance or consensus statement of kidney care in patients who underwent cardiac surgery.

## Figures and Tables

**Table 1:** Comparison of demographics and baseline clinical parameters across the stages of acute kidney injury.

Characteristic	No AKI	AKI Stage 1, Transient	AKI Stage 1, Persistent	AKI Stage 2 or 3, Transient	AKI Stage 2 or 3, Persistent	*p*-Value
Number of patients, *n*	1357 (67.5)	388 (19.3)	143 (7.1)	58 (2.9)	63 (3.1)	
Demographic data						
Age, years	64 (54–70)	65 (56–71)	63 (55–72)	63 (54–69)	65 (57–70)	0.127
Female, *n*	350 (25.8)	88 (22.7)	46 (32.2)	24 (41.4)	21 (33.3)	0.049
Body-mass index, kg/m^2^	23.9 (21.7–26.2)	24.0 (21.7–26.2)	24.4 (22.0–26.8)	23.3 (21.5–24.9)	23.8 (21.0–24.7)	0.150
Surgery type						
CABG, *n*	626 (46.1)	152 (39.2)	48 (33.6)	11 (19.0)	11 (17.5)	<0.001
Valvular heart surgery, *n*	644 (47.5)	213 (54.9)	86 (60.1)	43 (74.1)	47 (74.6)	<0.001
Thoracic aortic surgery, *n*	33 (2.4)	14 (3.6)	4 (2.8)	3 (5.2)	2 (3.2)	0.195
Combined surgery, *n*	54 (4.0)	9 (2.3)	5 (3.5)	1 (1.7)	3 (4.8)	0.347
Medical history						
Hypertension, *n*	647 (47.7)	214 (55.2)	80 (55.9)	35 (60.3)	32 (50.8)	0.004
Diabetes mellitus, *n*	341 (25.1)	108 (27.8)	34 (23.8)	14 (24.1)	15 (23.8)	0.926
Atrial fibrillation, *n*	166 (12.2)	70 (18.0)	22 (15.4)	12 (20.7)	14 (22.2)	<0.001
Cerebrovascular accident, *n*	132 (9.7)	38 (9.8)	21 (14.7)	14 (24.1)	9 (14.3)	0.005
COPD, *n*	80 (5.9)	15 (3.9)	15 (10.5)	1 (1.7)	1 (1.6)	0.335
Medication						
ACEi or ARB, *n*	236 (17.4)	57 (14.7)	24 (16.8)	8 (13.8)	14 (22.2)	0.741
β-blocker, *n*	255 (18.8)	69 (17.8)	18 (12.6)	4 (6.9)	10 (15.9)	0.026
Diuretics, *n*	167 (12.3)	49 (12.6)	20 (14.0)	10 (17.2)	8 (12.7)	0.180
Calcium channel blocker, *n*	203 (15.0)	59 (15.2)	21 (14.7)	11 (19.0)	5 (7.9)	0.625
Aspirin, *n*	677 (49.9)	210 (54.1)	87 (39.2)	19 (32.8)	20 (31.7)	0.041
Clopidogrel, *n*	255 (18.8)	72 (18.6)	27 (18.9)	6 (10.3)	8 (12.7)	0.182
Statins, *n*	346 (25.5)	89 (22.9)	37 (25.9)	12 (20.7)	13 (20.6)	0.173
Baseline laboratory findings						
Hematocrit, %	38.9 (35.3–42.5)	37.5 (33.8–41.3)	37.4 (33.8–40.1)	36.3 (32.4–40.7)	38.1 (34.6–40.0)	0.039
Serum creatinine, mg/dL	0.89 (0.75–1.00)	0.87 (0.70–1.03)	0.90 (0.76–1.04)	0.83 (0.66–0.95)	0.84 (0.76–0.94)	0.041
eGFR, mL/min/1.73 m^2^	87 (75–103)	90 (74–107)	85 (78–98)	91 (72–112)	89 (79–100)	0.044
Albumin, mg/dL	4.1 (3.9–4.4)	4.1 (3.8–4.4)	4.1 (3.8–4.4)	4.0 (3.6–4.3)	3.9 (3.6–4.3)	0.094
Operation and anesthesia details						
Operation time, hour	6.0 (5.2–6.9)	6.3 (5.4–7.6)	6.6 (5.5–7.7)	7.1 (6.1–8.5)	7.1 (5.8–8.7)	0.005
Crystalloid administration, mL/kg/h	6.0 (3.8–8.6)	5.2 (3.1–7.6)	4.7 (3.1–7.0)	4.5 (3.6–6.4)	5.3 (3.6–7.1)	0.010
Colloid administration, mL/kg/h	2.1 (0.8–3.9)	1.9 (0.7–3.6)	1.7 (0.8–3.1)	1.9 (1.4–2.4)	1.7 (0.9–2.6)	0.040
pRBC transfusion, units	2 (0–3)	2 (1–3)	2 (0–3)	2 (0–5)	2 (1–4)	0.014
FFP transfusion, units	0 (0–3)	0 (0–3)	2 (0–5)	3 (0–5)	2 (0–5)	<0.001
Intraoperative norepinephrine infusion, *n*	386 (28.4)	130 (33.5)	62 (43.4)	38 (65.5)	30 (52.4)	0.005
Intraoperative epinephrine infusion, *n*	71 (5.2)	31 (8.0)	17 (11.9)	6 (10.3)	10 (15.9)	0.001

Data were presented as median (interquartile range) for continuous data and number (%) for categorical variables. ACEi = angiotensin-converting enzyme inhibitor; ARB = angiotensin receptor blocker; CABG = coronary artery bypass surgery; COPD = chronic obstructive pulmonary disease; eGFR = estimated glomerular filtration rate; FFP = fresh frozen plasma; pRBC = packed red blood cells.

**Table 2:** Multivariable Cox regression analysis for new-onset chronic kidney disease or all-cause mortality during three years after cardiac surgery (*n* = 1356).

Variable	Hazard Ratio	95% CI	*p*-Value
Age, per 10 years	1.18	1.03–1.43	0.038
Female	1.12	0.65–1.63	0.696
Body mass index, kg/m^2^	1.13	1.06–1.33	0.001
History of hypertension	1.05	0.92–1.23	0.305
History of diabetes mellitus	1.19	1.06–1.41	0.045
Ischemic heart disease	1.03	0.55–2.10	0.942
Atrial fibrillation	1.20	0.66–1.98	0.642
Preoperative left ventricle ejection fraction, %	0.99	0.96–1.01	0.098
Preoperative hematocrit, %	0.98	0.95–1.01	0.175
Preoperative albumin, g/dL	0.92	0.76–1.22	0.250
Preoperative estimated glomerular filtration rate, mL/min/1.73 m^2^	0.85	0.83–0.87	<0.001
Postoperative acute kidney injury			
No acute kidney injury	baseline		
Acute kidney injury stage 1, transient, less than 48 h	1.95	0.83–3.02	0.246
Acute kidney injury stage 1, persistent, more than 48 h	3.11	2.62–4.91	<0.001
Acute kidney injury stage 2 or 3, transient, less than 48 h	4.07	2.98–6.11	<0.001
Acute kidney injury stage 2 or 3, persistent, more than 48 h	13.36	8.22–18.72	<0.001
Surgery type			
Valve replacement	baseline		
Coronary artery bypass graft	1.05	0.54–2.36	0.901
Aortic surgery	1.50	0.75–3.18	0.329
Combined procedures	2.05	0.71–5.18	0.152
Operation time, hour	1.05	0.88–1.65	0.435
Cardiopulmonary bypass time, hour	1.04	0.81–1.79	0.357
Intraoperative pRBC transfusion, unit	1.01	0.92–1.15	0.247
Intraoperative norepinephrine infusion	0.98	0.77–1.45	0.854
Intraoperative epinephrine infusion	1.09	0.84–1.33	0.432

CI = confidence interval; pRBC = packed red blood cell.

**Table 3:** Comparison of in-hospital and long-term outcomes across the stages of acute kidney injury.

Characteristic	No AKI	AKI Stage 1, Transient	AKI Stage 1, Persistent	AKI Stage 2 or 3, Transient	AKI Stage 2 or 3, Persistent	*p*-Value
Number of patients, *n*	1357 (67.5)	388 (19.3)	143 (7.1)	58 (2.9)	63 (3.1)	
AKI duration, days	-	2 (1–2)	5 (4–6) *	2 (2–2)	6 (5–7) *	<0.001
Hemodialysis during hospital stay, *n*	-	2 (0.5)	13 (9.1)	22 (37.9)	34 (54.0)	<0.001
Length of postoperative hospital stay, days	9 (7–13)	11 (8–16)	13 (9–19)*	15 (11–26)	18 (12–31) *	<0.001
Length of postoperative ICU stay, days	6 (3–9)	7 (4–10)	7 (4–12)	9 (7–14)	12 (6–20) *	<0.001
IABP insertion, *n*	70 (5.2)	24 (6.2)	15 (10.5)*	5 (8.6)	13 (27.1) *	<0.001
Reopen for surgical bleeding, *n*	21 (2.0)	10 (2.6)	6 (4.2)	3 (5.2)	6 (9.5)	0.021
Respiratory complications, *n*	178 (13.1)	73 (18.8)	28 (19.6)	14 (24.1)	11 (17.5)	0.001
In-hospital mortality, *n*	9 (0.7)	4 (1.0)	8 (5.6)*	2 (3.4)	12 (19.0) *	<0.001
eGFR < 60 mL/min/1.73 m^2^ at 3 months after surgery, *n*	43/1167 (3.7)	81/383 (21.1)	40/129 (31.0)	15/52 (28.8)	20/45 (44.4) *	<0.001
eGFR < 60 mL/min/1.73 m^2^ at 3 years after surgery, *n*	56/684 (8.2)	140/348 (40.2)	76/117 (65.0)*	17/28 (60.7)	26/31 (83.8) *	<0.001
Dependence on the hemodialysis at 3 years after surgery, *n*	8/684 (1.2)	10/348 (2.9)	12/117 (10.3)*	9/28 (32.1)	17/31 (54.8) *	<0.001

Data were presented as median (interquartile range) for continuous data and number (%) for categorical variables. AKI = acute kidney injury; eGFR = estimated glomerular filtration rate; ICU = intensive care unit; IABP = intra-aortic balloon pump. Respiratory complications include postoperative atelectasis, pneumonia, pulmonary edema, pleural effusion, and pneumothorax. *p*-values are the results of a one-way analysis of variance. * Significant difference between transient vs. persistent groups.

## Data Availability

The data presented in this study are available on request from the corresponding author.

## Supplementary Materials

The following are available online at https://www.mdpi.com/article/10.3390/jcm10081556/s1: Supplemental Figure S1: Flow diagram of the study. AKI = acute kidney injury, eGFR = estimated glomerular filtration rate, Supplemental Figure S2: Comparison of renal function measured by estimated glomerular filtration rate (eGFR) during three years after surgery according to the stages of acute kidney injury after cardiac or thoracic aortic surgery, Supplemental Figure S3: Follow up of renal function measured by estimated glomerular filtration rate (eGFR) of the patients who underwent cardiac or thoracic aortic surgery, Supplemental Table S1: Comparison of demographics and baseline clinical parameters across the stages of acute kidney injury, Supplemental Table S2: Comparison between included and excluded patients in the analysis of three years follow up of estimated glomerular filtration rate, Supplemental Table S3: Multivariable Cox regression analysis for new-onset chronic kidney disease during one year after cardiac surgery in all patients (*n* = 1789), Supplemental Table S4: Multivariable Cox regression analysis for new-onset chronic kidney disease during two years after cardiac surgery in all patients (*n* = 1491), Supplemental Table S5: Multivariable Cox regression analysis for new-onset chronic kidney disease during three years after cardiac surgery in all patients (*n* = 1356). Persistent acute kidney injury was defined with its duration of more than seven days.
