# Supplementary material for: Severity and Duration of Acute Kidney Injury and Chronic Kidney Disease after Cardiac Surgery
Source: J Clin Med. 2021 Apr 7;10(8):1556. doi: 10.3390/jcm10081556 (PMC8067973; doi:10.3390/jcm10081556)
Supplement: Supplementary file 1 [file jcm-10-01556-s001.pdf]

Severity and Duration of Acute Kidney Injury and Chronic Kidney Disease after Cardiac Surgery:

Supplemental Materials

Number	Title	Page
**Supplemental Figure S1**.	Flow diagram of the study.	2
**Supplemental Figure S2.**	Comparison of renal function measured by estimated glomerular filtration rate (eGFR) during 3 years after surgery according to the stages of acute kidney injury after cardiac or thoracic aortic surgery.	3
**Supplemental Figure S3.**	Follow-up of renal function measured by estimated glomerular filtration rate (eGFR) of the patients who underwent cardiac or thoracic aortic surgery.	4
**Supplemental Table S1**.	Baseline characteristics and perioperative parameters.	5
**Supplemental Table S2**.	Comparison of demographics and baseline clinical parameters between the patients with and without new-onset chronic kidney disease or all-cause mortality during three years after surgery.	7
**Supplemental Table S3**.	Comparison between included and excluded patients in the analysis of 3 years follow-up of estimated glomerular filtration rate.	9
**Supplemental Table S4**.	Multivariable Cox regression analysis for new-onset chronic kidney disease during one year after cardiac surgery in all patients (n = 1789).	11
**Supplemental Table S5**.	Multivariable Cox regression analysis for new-onset chronic kidney disease during two years after cardiac surgery in all patients (n = 1491).	13
**Supplemental Table S6**.	Multivariable Cox regression analysis for new-onset chronic kidney disease during three years after cardiac surgery in all patients (n = 1356). Persistent acute kidney injury was defined with its duration of more than 7 days.	14

Supplemental Figure S1. Flow diagram of the study. AKI = acute kidney injury, eGFR = estimated glomerular filtration rate.

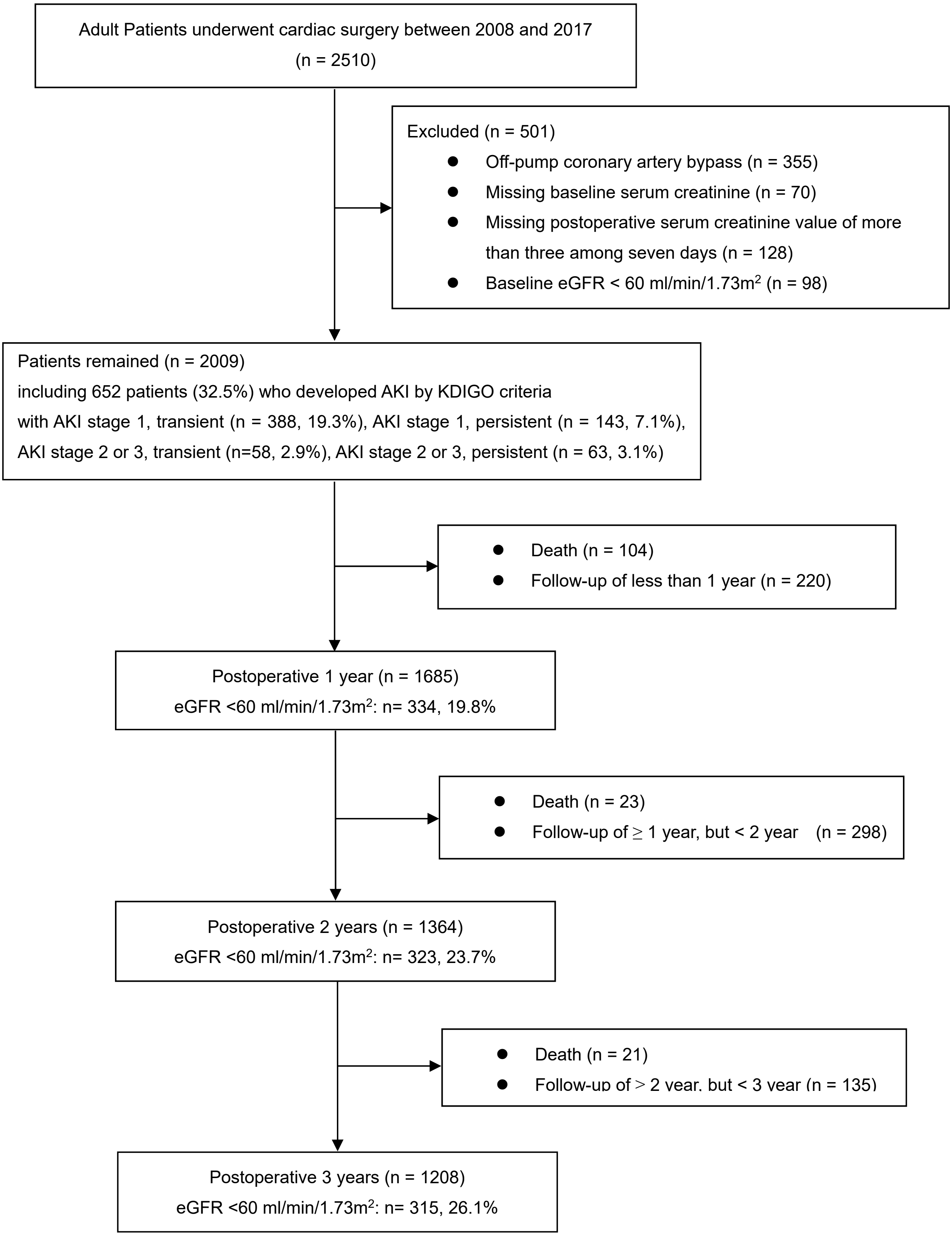

Supplemental Figure S2. Comparison of renal function measured by estimated glomerular filtration rate (eGFR) during 3 years after surgery according to the stages of acute kidney injury after cardiac or thoracic aortic surgery.

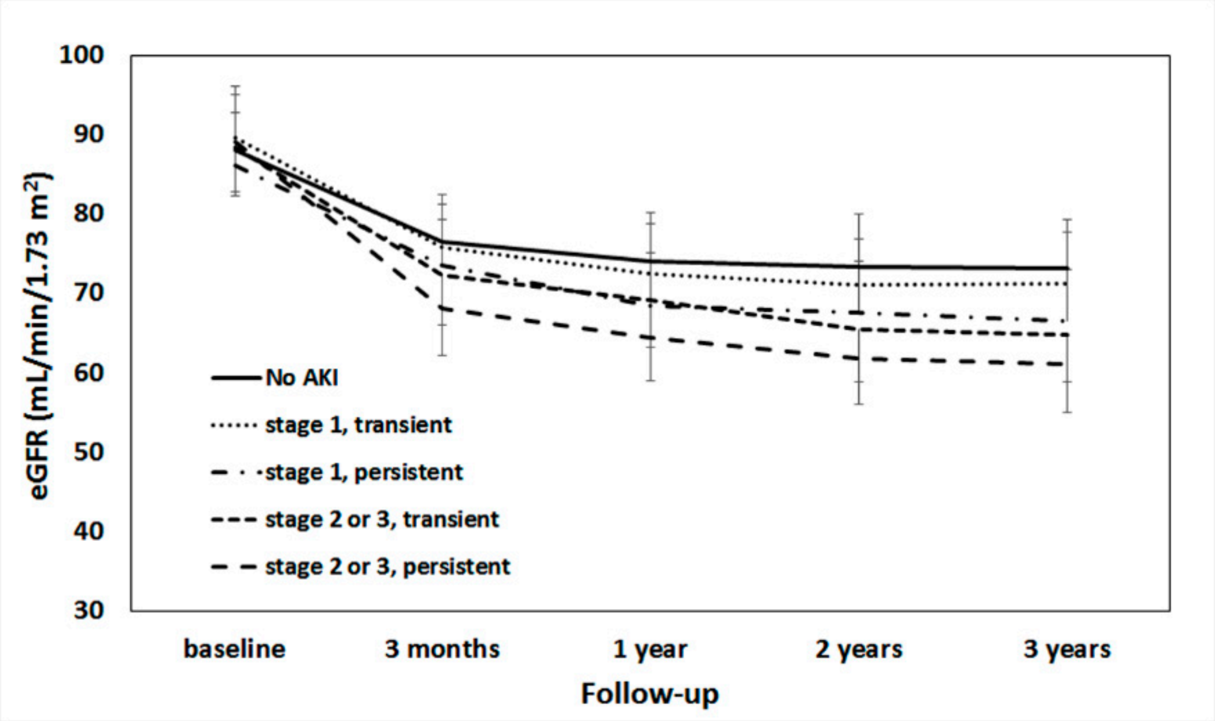

Supplemental Figure S3. Follow-up of renal function measured by estimated glomerular filtration rate (eGFR) of the patients who underwent cardiac or thoracic aortic surgery.

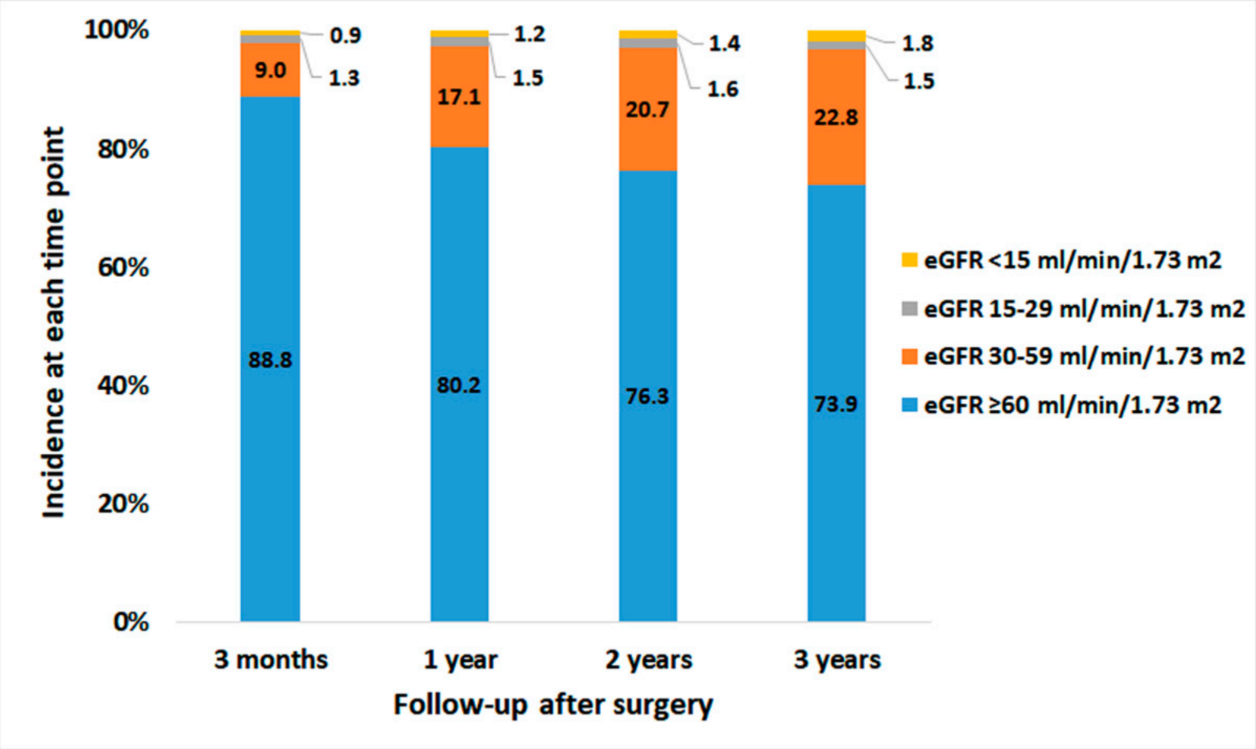

Supplemental Table S1. Baseline characteristics and perioperative parameters.

Characteristic	Value	Case without missing
Number of patients, n	2009	
Demographic data		
Age, years	63 (55 – 70)	100
Female, n	529 (26.3)	100
Body-mass index, kg/m^2	23.9 (21.7 – 26.1)	100
Surgery type		
CABG on pump, n	848 (42.2)	100
Valvular heart surgery, n	1033 (51.4)	100
Aortic valve replacement, n	415 (20.7)	100
Mitral valve replacement, n	459 (22.8)	100
Double valve replacement, n	129 (6.4)	100
Bentall operation, n	30 (1.5)	100
Thoracic aortic surgery, n	56 (2.8)	100
Total arch replacement, n	12 (0.6)	100
Ascending aorta replacement, n	44 (2.2)	100
Combined surgery, n	72 (3.6)	100
CABG with valve replacement, n	42 (2.1)	100
Valve replacement with aortic surgery, n	15 (0.7)	100
CABG with aortic surgery	15 (0.7)	100
Medical history		
Hypertension, n	1008 (50.2)	100
Diabetes mellitus, n	512 (25.5)	100
Atrial fibrillation, n	284 (14.1)	100
Cerebrovascular accident, n	214 (10.7)	100
COPD, n	112 (5.6)	100
Medication		
ACEi or ARB, n	339 (16.9)	100
β-blocker, n	356 (17.7)	100
Diuretics, n	254 (12.6)	100
Calcium channel blocker, n	299 (14.9)	100
Aspirin , n	946 (47.1)	100
Clopidogrel , n	368 (18.3)	100
Statins , n	497 (24.7)	100
Baseline laboratory findings		
Hematocrit, %	38.5 (34.7 – 42.0)	100
Serum creatinine, mg/dL	0.90 (0.75 – 1.01)	100
eGFR, mL/min/1.73 m^2	86 (74 – 103)	100
Albumin, g/dL	4.1 (3.9 – 4.4)	100
Operation and anesthesia details		
Operation time, hour	6.2 (5.3 – 7.2)	100
Cardiopulmonary bypass time, hour	4.4 (3.9 – 5.8)	100
Crystalloid administration, mL/kg/hr	5.5 (3.5 – 8.1)	100
Colloid administration, mL/kg/hr	1.9 (0.8 – 3.7)	100
pRBC transfusion, units	2 (0 – 3)	100
FFP transfusion, units	0 (0 – 3)	100
Intraoperative norepinephrine infusion, n	649 (32.3)	100
Intraoperative epinephrine infusion, n	135 (6.7)	100

Values are expressed as mean (SD), median [interquartile ranges] or number (%). ACEi =Angiotensin converting enzyme inhibitor; ARB = Angiotensin receptor blocker; CABG = Coronary artery bypass surgery; COPD = chronic obstructive pulmonary disease; eGFR = estimated glomerular filtration rate; FFP = fresh frozen plasma; pRBC = packed red blood cells.

Supplemental Table S2. Comparison of demographics and baseline clinical parameters between the patients with and without new-onset chronic kidney disease or all-cause mortality during three years after surgery.

Characteristic	Chronic kidney disease	No chronic kidney disease	P-value
Number of patients, n	463 (23.0)	1546 (77.0)	
Demographic data			
Age, years	65 (57 – 72)	63 (54 – 70)	<0.001
Female, n	138 (29.8)	391 (25.3)	0.053
Body-mass index, kg/m^2	24.1 (21.6 – 26.6)	23.9 (21.7 – 26.0)	0.089
Surgery type			<0.001
CABG, n	154 (33.3)	694 (44.9)	
Valvular heart surgery, n	275 (59.4)	758 (49.0)	
Thoracic aortic surgery, n	15 (3.2)	41 (2.7)	
Combined surgery, n	19 (4.1)	53 (3.4)	
Medical history			
Hypertension, n	247 (53.3)	761 (49.2)	0.120
Diabetes mellitus, n	119 (25.7)	393 (25.4)	0.903
Atrial fibrillation, n	78 (16.8)	206 (13.3)	0.056
Cerebrovascular accident, n	66 (14.3)	148 (9.6)	0.004
COPD, n	23 (5.0)	89 (5.8)	0.516
Medication			
ACEi or ARB, n	79 (17.1)	260 (16.8)	0.902
β-blocker, n	79 (17.1)	277 (17.9)	0.673
Diuretics, n	71 (15.3)	183 (11.8)	0.051
Calcium channel blocker, n	73 (15.8)	226 (14.6)	0.543
Aspirin , n	210 (45.3)	736 (47.6)	0.354
Clopidogrel , n	88 (19.0)	280 (18.1)	0.634
Statins , n	101 (21.8)	396 (25.6)	0.096
Baseline laboratory findings			
Hematocrit, %	37.4 (33.7 – 40.9)	38.7 (35.1 – 42.4)	<0.001
Serum creatinine, mg/dL	1.00 (0.81 – 1.11)	0.87 (0.74 – 0.99)	<0.001
eGFR, mL/min/1.73 m^2	75.3 (69.9 – 89.8)	89.5 (77.8 – 105.5)	<0.001
Albumin, mg/dL	4.1 (3.8 – 4.4)	4.1 (3.9 – 4.4)	0.212
Operation and anesthesia details			
Operation time, hour	6.6 (5.4 – 8.0)	6.0 (5.2 – 7.0)	<0.001
Crystalloid administration, mL/kg/hr	5.8 (3.6 – 8.3)	4.8 (3.0 – 7.5)	<0.001
Colloid administration, mL/kg/hr	2.1 (0.8 – 3.9)	1.7 (0.7 – 3.3)	0.002
pRBC transfusion, units	2 (0 – 3)	2 (0 – 3)	0.10
FFP transfusion, units	1 (0 – 3)	0 (0 – 3)	<0.001
Intraoperative norepinephrine infusion, n	190 (41.0)	459 (29.7)	<0.001
Intraoperative epinephrine infusion, n	45 (9.7)	90 (5.8)	0.003

Data were presented as median (interquartile range) for continuous data and number (%) for categorical variables. ACEi =Angiotensin converting enzyme inhibitor; ARB = Angiotensin receptor blocker; CABG = Coronary artery by-pass surgery; COPD = chronic obstructive pulmonary disease; eGFR = estimated glomerular filtration rate; FFP = fresh frozen plasma; pRBC = packed red blood cells.

Supplemental Table S3. Comparison between included and excluded patients in the analysis of 3 years follow-up of estimated glomerular filtration rate.

Characteristic	Included patients	Excluded patients	P-value
Number of patients, n	1208 (60.1)	801 (39.9)	
Demographic data			
Age, years	63 (55 – 70)	63 (54 – 71)	0.969
Female, n			
Body-mass index, kg/m^2	23.9 (21.9 – 26.2)	23.8 (21.7 – 26.0)	0.154
Surgery type			
CABG under CPB, n	493 (40.8)	355 (44.3)	0.119
Valvular heart surgery, n	639 (52.9)	394 (49.2)	0.103
Aortic valve replacement, n	263 (21.8)	152 (19.0)	
Mitral valve replacement, n	284 (23.5)	175 (21.8)	
Double valve replacement, n	77 (6.4)	52 (6.5)	
Bentall operation, n	15 (1.2)	15 (1.9)	
Thoracic aortic surgery, n	39 (3.2)	17 (2.1)	0.140
Total arch replacement, n	9 (0.7)	3 (0.4)	
Ascending aorta replacement, n	30 (2.5)	14 (1.7)	
Combined surgery, n	37 (3.1)	35 (4.4)	0.123
CABG with valve replacement, n	26 (2.2)	16 (2.0)	
Valve replace with aortic surgery, n	6 (0.5)	9 (1.1)	
CABG with aortic surgery	5 (0.4)	10 (1.2)	
Medical history			
Hypertension, n	603 (49.9)	405 (50.6)	0.777
Diabetes mellitus, n	310 (25.7)	202 (25.2)	0.823
Atrial fibrillation, n	173 (14.3)	111 (13.9)	0.770
Cerebrovascular accident, n	130 (10.8)	84 (10.5)	0.845
COPD, n	55 (4.6)	57 (7.1)	0.019
Medication			
ACEi or ARB, n	205 (17.0)	134 (16.7)	0.888
β-blocker, n	203 (16.8)	153 (19.1)	0.187
Diuretics, n	136 (11.3)	118 (14.7)	0.022
Calcium channel blocker, n	155 (12.8)	144 (18.0)	0.002
Aspirin , n	577 (48.8)	369 (46.1)	0.455
Clopidogrel , n	221 (18.3)	147 (18.4)	0.974
Statins , n	294 (24.3)	203 (25.3)	0.609
Baseline laboratory findings			
Hematocrit, %	38.5 (34.9 – 42.0)	38.5 (34.6 – 42.0)	0.714
eGFR, mL/min/1.73 m^2	86 (77 – 102)	86 (75 – 103)	0.645
Serum creatinine, mg/dL	0.90 (0.75 – 1.01)	0.90 (0.79 – 1.00)	0.561
Albumin, mg/dL	4.1 (3.9 – 4.4)	4.1 (3.8 – 4.4)	0.126
Operation and anesthesia details			
Operation time, hour	6.1 (5.3 – 7.2)	6.2 (5.1 – 7.2)	0.347
Crystalloid administration, mL/kg/hr			0.428
Colloid administration, mL/kg/hr			0.139
pRBC transfusion, units	2 (0 – 3)	2(0 – 3)	0.780
FFP transfusion, units	0 (0 – 3)	0 (0 – 3)	0.098
Intraoperative norepinephrine infusion, n	397 (32.9)	252 (31.5)	0.510
Intraoperative epinephrine infusion, n	71 (5.9)	64 (8.0)	0.064

Values are expressed as mean (SD), median [interquartile ranges] or number (%).

ACEi =Angiotensin converting enzyme inhibitor; ARB = Angiotensin receptor blocker; CABG = Coronary artery bypass surgery; COPD = chronic obstructive pulmonary disease; CPB = cardiopulmonary bypass; eGFR = estimated glomerular filtration rate; FFP = fresh frozen plasma; pRBC = packed red blood cells.

Supplemental Table S4. Multivariable Cox regression analysis for new-onset chronic kidney disease during one year after cardiac surgery in all patients (n = 1789).

Variable	Hazard Ratio	95% CI	*P*-value
Age, per 10 years	1.04	1.01 – 1.08	0.043
Female	0.89	0.61 – 1.30	0.548
Body-mass index, kg/m^2	1.04	0.99 – 1.08	0.141
History of hypertension	1.07	0.90 – 1.34	0.249
History of diabetes mellitus	1.12	0.92 – 1.41	0.117
Ischemic heart disease	1.13	0.66 – 1.93	0.659
Atrial fibrillation	1.21	0.80 – 1.82	0.372
Preoperative left ventricle ejection fraction, %	0.98	0.97 – 0.99	0.027
Preoperative hematocrit, %	0.98	0.95 – 1.01	0.199
Preoperative albumin, g/dL	0.99	0.72 – 1.38	0.991
Preoperative estimated glomerular filtration rate, ml/min/1.73m^2	0.96	0.95 – 0.97	<0.001
Postoperative acute kidney injury			
No acute kidney injury	baseline		
Acute kidney injury stage 1, transient, less than 48 hours	2.41	0.72 – 5.41	0.294
Acute kidney injury stage 1, persistent, more than 48 hours	3.91	2.82 – 5.66	<0.001
Acute kidney injury stage 2 or 3, transient, less than 48 hours	4.76	3.05 – 6.37	<0.001
Acute kidney injury stage 2 or 3, persistent, more than 48 hours	17.49	8.70 – 35.18	<0.001
Surgery type			
Valve replacement	baseline		
Coronary artery bypass graft	1.16	0.66 – 2.03	0.603
Aortic surgery	1.30	0.60 – 2.78	0.507
Combined procedures	1.76	0.77 – 4.04	0.182
Operation time, hour	1.06	0.81 – 1.79	0.645
Cardiopulmonary bypass time, hour	1.03	0.75 – 1.88	0.476
Intraoperative pRBC transfusion, unit	1.01	0.88 – 1.26	0.765
Intraoperative norepinephrine infusion	0.97	0.71 – 1.59	0.846
Intraoperative epinephrine infusion	1.07	0.80 – 1.38	0.654

CI = confidence interval.

Supplemental Table S5. Multivariable Cox regression analysis for new-onset chronic kidney disease during two years after cardiac surgery in all patients (n = 1491).

Variable	Hazard Ratio	95% CI	*P*-value
Age, per 10 years	1.10	1.02 – 1.29	0.029
Female	0.99	0.62 – 1.58	0.973
Body-mass index, kg/m^2	1.08	1.02 – 1.14	0.006
History of hypertension	1.10	0.78 – 1.58	0.561
History of diabetes mellitus	1.22	1.08 – 1.35	0.044
Ischemic heart disease	1.02	0.49 – 1.87	0.910
Atrial fibrillation	1.42	0.86 – 2.34	0.168
Preoperative left ventricle ejection fraction, %	0.98	0.97 – 1.02	0.075
Preoperative hematocrit, %	0.99	0.96 – 1.03	0.584
Preoperative albumin, g/dL	1.09	0.74 – 1.63	0.641
Preoperative estimated glomerular filtration rate, ml/min/1.73m^2	0.91	0.89 – 0.92	<0.001
Postoperative acute kidney injury			
No acute kidney injury	baseline		
Acute kidney injury stage 1, transient, less than 48 hours	1.94	0.89 – 3.37	0.137
Acute kidney injury stage 1, persistent, more than 48 hours	3.83	2.60 – 5.64	<0.001
Acute kidney injury stage 2 or 3, transient, less than 48 hours	4.60	3.09 – 6.96	<0.001
Acute kidney injury stage 2 or 3, persistent, more than 48 hours	16.04	7.95 – 33.22	<0.001
Surgery type			
Valve replacement	baseline		
Coronary artery bypass graft	1.36	0.68 – 2.70	0.381
Aortic surgery	0.97	0.38 – 2.48	0.947
Combined procedures	1.58	0.93 – 5.12	0.068
Operation time, hour	1.04	0.86 – 1.77	0.514
Cardiopulmonary bypass time, hour	1.02	0.80 – 1.88	0.614
Intraoperative pRBC transfusion, unit	1.02	0.93 – 1.17	0.457
Intraoperative norepinephrine infusion	0.95	0.69 – 1.51	0.742
Intraoperative epinephrine infusion	1.06	0.82 – 1.37	0.543

CI = confidence interval.

Supplemental Table S6. Multivariable Cox regression analysis for new-onset chronic kidney disease during three years after cardiac surgery in all patients (n = 1356). Persistent acute kidney injury was defined with its duration of more than 7 days.

Variable	Hazard Ratio	95% CI	*P*-value
Age, per 10 years	1.17	1.04 – 1.52	0.042
Female	1.10	0.66 – 1.70	0.741
Body-mass index, kg/m^2	1.11	1.07 – 1.35	0.004
History of hypertension	1.07	0.94 – 1.27	0.415
History of diabetes mellitus	1.16	1.06 – 1.40	0.006
Ischemic heart disease	1.06	0.57 – 2.12	0.847
Atrial fibrillation	1.22	0.68 – 1.94	0.548
Preoperative left ventricle ejection fraction, %	0.99	0.95 – 1.01	0.120
Preoperative hematocrit, %	0.98	0.96 – 1.01	0.189
Preoperative albumin, g/dL	0.93	0.77 – 1.22	0.215
Preoperative estimated glomerular filtration rate, ml/min/1.73m^2	0.84	0.83 – 0.88	<0.001
Postoperative acute kidney injury			
No acute kidney injury	baseline		
Acute kidney injury stage 1, less than 7 days	2.46	1.45 – 3.47	<0.001
Acute kidney injury stage 1, more than 7 days	3.85	2.89 – 5.63	<0.001
Acute kidney injury stage 2 or 3, less than 7 days	8.45	3.75 – 11.24	<0.001
Acute kidney injury stage 2 or 3, more than 7 days	15.75	9.42 – 24.19	<0.001
Surgery type			
Valve replacement	baseline		
Coronary artery bypass graft	1.06	0.54 – 2.33	0.842
Aortic surgery	1.52	0.77 – 3.17	0.451
Combined procedures	2.06	0.72 – 5.17	0.201
Operation time, hour	1.06	0.90 – 1.66	0.421
Cardiopulmonary bypass time, hour	1.06	0.80 – 1.82	0.264
Intraoperative pRBC transfusion, unit	1.01	0.91 – 1.16	0.254
Intraoperative norepinephrine infusion	0.99	0.78 – 1.40	0.749
Intraoperative epinephrine infusion	1.10	0.85 – 1.33	0.411

CI = confidence interval.

Supplemental Table S7. Multivariable logistic regression analysis for persistent stage 2 or 3 acute kidney injury or acute kidney injury requiring hemodialysis (n=65).

Variable	Hazard Ratio	95% confidence interval	*P*-value
Age, per 10 years	1.13	1.03 – 1.58	0.010
History of hypertension	1.28	1.06 – 1.69	0.008
Atrial fibrillation	1.15	0.86 – 1.86	0.343
Preoperative hematocrit, %	0.94	0.84 – 0.99	0.046
Preoperative albumin, g/dL	0.93	0.81 – 1.31	0.350
Surgery type			
Valve replacement	baseline		
CABG, on pump	1.11	0.78 – 1.92	0.603
Aortic surgery	1.33	0.95 – 2.24	0.080
Combined procedures	1.74	1.00 – 3.84	0.048
Operation time, hour	1.07	0.94 – 1.29	0.247
Cardiopulmonary bypass time, hour	1.03	0.96– 1.33	0.154
Intraoperative pRBC transfusion, unit	1.01	1.10 – 2.38	0.039
Intraoperative norepinephrine infusion	0.97	0.75 – 3.41	0.846
Intraoperative epinephrine infusion	1.07	1.11 – 2.95	0.035

CABG = coronary artery bypass graft, pRBC = packed red blood cell.
